# Peripheral blood lymphopenia in sarcoidosis associates with *HLA-DRB1* alleles but not with lung immune cells and organ involvement

**DOI:** 10.1093/cei/uxad052

**Published:** 2023-05-10

**Authors:** Pernilla Darlington, Jonas Melin, Natalia Rivera, Johan Grunewald, Anders Eklund, Susanna Kullberg

**Affiliations:** Department of Internal Medicine, Södersjukhuset, Sweden; Department of Clinical Science and Education, Södersjukhuset and Karolinska Institutet, Stockholm, Sweden; Department of Internal Medicine, Södersjukhuset, Sweden; Respiratory Medicine Division, Department of Medicine, Karolinska Institutet, Stockholm, Sweden; Respiratory Medicine Division, Department of Medicine, Karolinska Institutet, Stockholm, Sweden; Department of Respiratory Medicine, Theme Inflammation and Ageing, Karolinska University Hospital, Stockholm, Sweden; Respiratory Medicine Division, Department of Medicine, Karolinska Institutet, Stockholm, Sweden; Department of Respiratory Medicine, Theme Inflammation and Ageing, Karolinska University Hospital, Stockholm, Sweden; Respiratory Medicine Division, Department of Medicine, Karolinska Institutet, Stockholm, Sweden; Department of Respiratory Medicine, Theme Inflammation and Ageing, Karolinska University Hospital, Stockholm, Sweden

**Keywords:** sarcoidosis, lymphopenia, bronchoalveolar lavage

## Abstract

Different human leukocyte antigen (HLA) alleles associate with disease phenotypes in sarcoidosis. Peripheral blood (PB) lymphopenia is reported as more common in sarcoidosis patients with worse prognosis. The mechanisms behind are unrecognized but a PB depletion due to lymphocytes migrating to lung and/or extra pulmonary organs has been suggested. Insights into associations between HLA alleles, lung immune cells, clinical phenotype including extra pulmonary manifestations (EPM), and PB lymphopenia may provide mechanistic clues and enable adequate intervention in this patient group. In this situdy,141 treatment naïve, newly diagnosed patients were retrospectively identified in a Swedish cohort of sarcoidosis patients. Data on *HLA-DRB1* alleles, lung immune cells from bronchoalveolar lavage fluid (BALF), PB lymphocytes and clinical parameters including treatment and disease course (chronic vs. resolving) were collected. The patients were followed for 2 years. PB lymphopenia associated with male sex, development of non-resolving disease, a need for first- and second-line systemic immunosuppressant treatment and HLA- DRB1*07. No correlation between BALF and PB lymphocytes, and no difference in EPM was detected between patients with and without PB lymphopenia. In conclusion, PB lymphopenia is associated with a more severe disease phenotype and carriage of the HLA-DRB1*07 allele. The results do not lend support to the hypothesis about sarcoidosis PB lymphopenia being due to a migration of PB lymphocytes to other organs. Rather, they provide a basis for future studies on the connection between HLA-DRB1*07 and PB lymphopenia mechanisms.

## Background

The clinical presentation of the inflammatory systemic disease sarcoidosis is variable. Virtually any organ can be affected, but the lungs and/or intrathoracic lymph nodes are engaged in most cases. Patients with Löfgren´s syndrome (LS) experience an acute and often self-limiting disease, while patients with non-Löfgren´s syndrome (non-LS) more often present with a slower developing and non-resolving disease. There is no cure and despite treatment with first- and second-line therapy (usually corticosteroids and methotrexate), many patients disclose a progressive disease course leading to organ function impairments and sometimes failure [1].

The exact immunological events leading to the sarcoid inflammation remains unknown. It has been established, though, that both genetic factors and a dysregulated immune system, characterized by an excess of activated CD4+ T cells in the lungs, are involved [1]. As opposed to the T-cell alveolitis in the lungs, peripheral blood (PB) lymphopenia was reported already in the 70s in a subset of sarcoidosis patients [2, 3].

Especially, the human leukocyte antigen (HLA) Class II, but also HLA Class I and Class III genes as well as a number of non-HLA genes have been associated with disease risk and phenotype. Some of these genes vary by ancestry [4–6], while some variants seem similar between different ethnic cohorts. HLA-DRB1*1101 is reported as risk factor for sarcoidosis in both blacks and whites [4]. However, interpretation of genetic data is complicated by some data from similar ethnic cohorts having provided conflicting results [4, 6]. In Swedish patients, HLA-DRB1*04 is associated with extra pulmonary manifestations, HLA-DRB1*03 with Löfgren’s syndrome and resolving disease, while HLA-DRB1*15 is more frequent in patients with a chronic disease course [7, 8]. Furthermore, high expression of the CD4+ T-cell receptor segment Vα2.3 (TRAV2.3) has been connected to a good prognosis [9].

PB lymphopenia seems more common in patients with less favorable prognosis and high inflammatory activity but the mechanisms behind are not fully understood [3, 10, 11]. Migration of PB lymphocytes to the lung [12] as well as a lymphocyte depletion due to antibody mediated depletion [13] have been suggested as possible explanations. Insight into the influence of *HLA-DRB1* alleles, bronchoalveolar fluid (BALF) cells and patient phenotype on PB lymphopenia may provide mechanistic clues and help to disentangle immunological mechanisms behind. This study was designed to assess the association of *HLA-DRB1* alleles, clinical characteristics and BALF cells with PB lymphopenia, in patients with sarcoidosis.

## Materials and methods

### Study subjects

A cohort of approximately 2000 patients with sarcoidosis from a local registry at Karolinska University Hospital, Stockholm Sweden, containing clinical and genotype data was retrospectively searched for consecutive patients included between May 2015 to June 2019. Using criteria outlined by the World Association of Sarcoidosis and other Granulomatous Disorders (WASOG), subjects were diagnosed through typical clinical and radiographic manifestations, findings at bronchoscopy including elevated CD4/CD8 cell ratio and positive biopsies [14]. Inclusion was restricted to newly diagnosed, treatment-naïve patients with non-LS. The majority was of European ancestry. Included patients were divided into two groups; one with PB lymphopenia and one without PB lymphopenia. All included patients had signed a written consent form according to Declaration of Helsinki, and approval was granted from the regional ethical review board.

### Parameters

PB lymphopenia was defined using laboratory reference values. For 67 patients, this was defined as lymphocytes <1.0 × 10^9^/L. Due to changes in reference values during the study period and the use of external laboratories, it was defined as <1.1 × 10^9^/L for 55 patients and <0.8 × 10^9^/L for 19 patients.

Data on sex, age at sarcoidosis diagnosis, *HLA-DRB1* alleles, serum angiotensin-converting enzyme (s-ACE), extra pulmonary manifestations (EPM), and BALF data were collected. BALF data included total number of cells (×10^6^), cell concentration (×10^6^/L), percentages (%) and concentrations (×10^6^/L) of macrophages and lymphocytes, CD4/CD8 ratio and percentage of TRAV2.3+ CD4+ T cells. PB lymphocytes were matched as close as possible to the date of BAL. Information about first- and second-line treatment with systemic corticosteroids and cytotoxic agents, respectively, within 2 years from diagnosis was extracted from the medical record. EPM was evaluated as numbers of organs involved and defined as a positive biopsy from the affected organ or obvious symptoms/assessment from a specialist in the area. Enlarged lymph nodes were regarded as one organ. Remaining signs of disease >2 years were defined as non-resolving disease, evaluated by chest X-ray, lung function test, presence of EPM, patient symptoms and laboratory signs of inflammation.

### HLA typing

Genomic DNA was extracted from whole blood samples. *HLA-DRB1* allelic groups were determined using the PCR-SSP technique with SSO DR low-resolution kit according to the manufacturers One Lambda’s recommendations.

### Bronchoscopy

Bronchoscopy with BAL was performed as previously described [15]. Cells in BALF were separated from recovered fluid by centrifugation, fixed on cytospin slides and stained with Giemsa for calculation of leucocyte differential count. Surface markers expressed on T cells were analyzed with flow cytometry using FACS CANTO II flow cytometer (BD Bioscience). Data were processed using a FACS Diva 6.1.2 software (BD Bioscience). The antibody used as surface marker for TRAV2.3+ CD4+ T cells was Vα2.3 (Thermo Scientific).

### Statistical analysis

The Mann-Whitney test was used for comparisons between the lymphopenia and non-lymphopenia groups and Spearman´s rank for correlations. *P*-value significance was set at <0.05. To minimize the risk for type 1 error when comparing the 13 different *HLA-DRB1* allele frequencies Bonferroni correction *P*-value (*P* < 0.05/13 = *P* < 0.0038) was taken into consideration. *P*-values are presented uncorrected.

The free software Jamovi 1.1.9.0 (https://www.jamovi.org) were used for all statistical analysis.

## Results

### Study subjects

A total of 172 patients were reviewed and 141 fulfilled criteria for inclusion. The majority were male and disclosed a chronic disease. Of 53 patients treated within 2 years, 49 received prednisone, 2 methotrexate, 1 hydroxychloroquine, and 1 chloroquine phosphate as first-line therapy. All 18 patients that were treated with second-line received methotrexate, except one that instead received azathioprine. Prednisone had been given to all patients that later received second-line therapy. Median number of days between BAL and PB sampling was 30 days (25th-75th percentile, 20-47) and mean number of days 40 (standard deviation 44). For details on study subjects, see Table 1.

**Table 1.** Patient characteristics

Parameter	All patients	Lymphopenia	No lymphopenia	*P*-value
Patients	141	41 (29)	100 (71)	
Male/female	100/41 (71/29)	37/4 (90/10)	63/37 (63/27)	0.001
Age (years)	46 (39-56)	49 (39-55)	46 (38-56)	0.353
Elevated s-ACE^1^	40 (30)	13 (35)	27 (28)	0.455
Chronic disease^2^	116 (93)	36 (100)	80 (90)	<0.05
First line	53 (38)	25 (61)	28 (28)	<0.001
Second line	18 (13)	12 (29)	6 (6)	<0.001
Patients with EPM	62 (44)	18 (44)	44 (44)	0.994
Numbers of EPM 0/1/2/3/4/5	81/38/15/4/2/1	23/13/5/0/0/0	58/25/10/4/2/1	ns/ns/ns/ns/ns/ns
Scadding stage 0/I/II/III/IV	5/39/74/16/7	0/9/22/5/5	5/30/52/11/2	ns/ns/ns/ns/*P* < 0.05

Lymphopenia and no lymphopenia denote patient groups with and without lymphopenia, respectively.

^1^As nine patients were excluded from the analysis of s-ACE due to treatment with ACE inhibitors, 132 patients were included in the analysis and 40 of these disclosed elevated s-ACE.

^2^Sixteen patients were excluded from chronicity analysis as follow-up data was lacking, 116 out of included 125 had a chronic disease. First line and second line denote patients treated with first- and second-line treatments, respectively. Data is presented as *n* (%) or median (25th–75th percentile).

### Lymphopenia

Lymphopenia at diagnosis was present in 41 patients. Details of lymphopenia and non-lymphopenia groups are shown in Table 1. In brief, lymphopenia was more common among men. The lymphopenia group also presented more often with fibrotic lung infiltrates (stage IV). All patients with lymphopenia disclosed a non-resolving disease and 61% received systemic treatment within the first 2 years compared to 28% in the no lymphopenia group. Second-line treatment had been given to 29% and 6% of patients with and without lymphopenia, respectively. No difference was observed between lymphopenia and no lymphopenia patients neither regarding presence (yes or no), nor numbers of EPM.

### BALF data

As shown in Table 2, there was no difference in any of the examined BALF parameters between the lymphopenia and no lymphopenia groups. No correlation between number of PB lymphocytes and any of the examined BALF parameters was observed (not shown).

**Table 2.** BALF data

BALF parameter	Lymphopenia	No lymphopenia	p-value
Total number of cells (×10^6^)	21 (14-37)	22 (16-35)	0.7
Total cell concentration (×10^6^/L)	155 (113-237)	186 (136-226)	0.6
Macrophages (×10^6^/L)	110 (90-173)	123 (92-158)	0.6
Macrophages (%)	71 (58-79)	75 (60-82)	0.3
Lymphocytes (×10^6^/L)	37 (23-83)	40 (25-66)	0.9
Lymphocytes (%)	25 (18-41)	23 (15-36)	0.3
CD4/CD8	6.4 (3.7-9.3)	6.6 (4.4-9.1)	0.8
TRAV2.3+ (%)	4.2 (2.7-6.1)	4.0 (3.4-7.5)	0.4

Lymphopenia and no lymphopenia denote patient groups with and without lymphopenia, respectively. TRAV2.3+ (%) denotes percentage of CD4+ T cells expressing TRAV2.3. Data was available from 116 patients and is presented as median (25^th^-75^th^ percentile).

### HLA-DRB1 alleles

**Figure 1.**
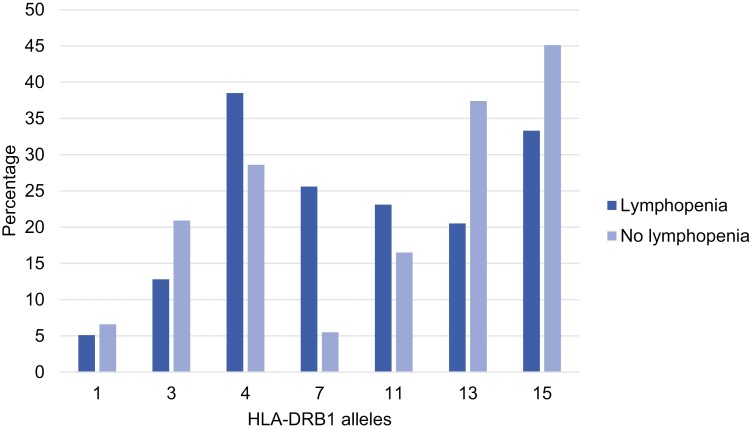
Frequency of HLA-DRB1 alleles amongst lymphopenia (*n* = 39) and no lymphopenia groups (*n* = 91). Eleven patients were not HLA typed (2 in the lymphopenia and 9 in the no lymphopenia group)

HLA DRB1*07 was more common in patients with lymphopenia than in patients without lymphopenia (*P* = 0.001), 67% of HLA-DRB1*07 carriers had lymphopenia at diagnosis. As shown in Fig. 1, HLA DRB1*04 was the most frequent allele in the lymphopenia group, but the frequency of 38% was not significantly different compared to 29% in the no lymphopenia group.

## Discussion

In this study, we found an association with PB lymphopenia in patients from a Swedish cohort with newly diagnosed sarcoidosis and male sex, more advanced radiographic stage, development of non-resolving disease, a need for systemic immunosuppressant treatment including second-line treatment and HLA-DRB1*07.

A correlation between PB lymphopenia and less favorable prognosis is in line with previous results. Already in the 70´s Böttger reported poorer long-time prognosis of sarcoidosis patients with low absolute numbers of PB lymphocytes [10], and Selroos and Koivunen found decreased PB lymphocyte values in patients requiring treatment with corticosteroids [3]. Later, Sweiss et al. demonstrated that the PB lymphopenia in sarcoidosis patients was more common in patients with severe organ involvement and was not related to medical therapy, suggesting that lymphopenia may relate more to disease pathology than medical therapy [11].

Still, the mechanisms of PB lymphopenia in sarcoidosis are unclear. Our finding of lymphopenia already at diagnosis in treatment-naïve patients lend support to the hypothesis that lymphopenia is related to sarcoidosis disease mechanisms, and not to treatment. Baughman and Hurtubise found a correlation between low PB CD4+ T-lymphocytes and high BALF CD4+ T-lymphocytes and Sweiss et al. found an association between severe organ involvement and PB lymphopenia, speaking for a migration of these cells and subsequent sequestration in the lung and/ or extra pulmonary organs [11, 12].

However, in this study we did not detect a correlation between BALF and PB lymphocytes, and no difference in presence of EPM between lymphopenia and no lymphopenia groups. It is well established that the lung lymphocytosis in sarcoidosis patients is attributed to a CD4 T-lymphocyte expansion. However, the PB lymphopenia seems not only be attributed to a decrease of CD4+ T-lymphocytes, but also a decrease of CD8+ and CD19+ lymphocytes, the latter representing B-cells [11].

Interestingly, a more recent study found an association with PB lymphopenia in sarcoidosis and under expression of certain small noncoding microRNAs acting as post-transcriptional immune modulators involved in apoptotic pathways and genes related to lymphopenia [16]. Also, already 1973 results from an *in vitro* study showed decreased survival of lymphocytes from sarcoidosis patients compared to normal controls [17]. A follow-up study demonstrated an increase of immunoglobulin-bearing lymphocytes, especially in patients with disseminated or advanced disease, which might contribute to damage and removal by the reticuloendothelial system. Subsequently, alterations in lymphocyte differentiation, proliferation, function and survival may be another plausible mechanism explaining the PB lymphopenia in sarcoidosis. A few studies investigated a possible influence of *HLA-DRB1* alleles on lymphocyte homeostasis. In for instance Colombian patients with systemic lupus erythematosus and German patients with rheumatoid arthritis, PB lymphopenia was associated with specific *HLA-DRB1* alleles [18, 19]. A recent Swedish study showed that sarcoidosis associated genetic variants influenced quantitative levels of BALF immune cells [20]. Thus, we think our finding that the majority of HLA-DRB1*07 positive patients belonged to the PB lymphopenia group deserves further attention. For instance, a possible association with genes involved in lymphocyte biology and apoptotic pathways needs to be investigated.

Furthermore, we found that most patients with PB lymphopenia were men suggesting that also hormonal factors can be involved in development of lymphopenia. This is in line with previous results demonstrating differences in disease manifestations and disease course between men and women [21–23].

Patients with lymphopenia in our study had more often received 1^st^ (i.e. corticosteroids) and 2^nd^ line treatment compared to patients without lymphopenia indicating that the PB lymphopenia sarcoidosis phenotype is not only associated with a need for treatment, but also resistant to systemic corticosteroids. Interestingly, CD4+ lymphopenia in PB has been associated with resistance to conventional immunosuppressant such as corticosteroids and methotrexate but responsive to treatment with TNF-α inhibitors. Anti TNF-α treatment in this patient group led to an increase in absolute PB lymphocyte, CD4+ T-lymphocyte and CD19+ B-lymphocyte counts, suggesting TNF-α being of mechanistic importance [24, 25]. The sarcoid inflammation has, at least partly, been explained by dysfunctional T regulatory cells (T_reg_) [1, 26]. T_reg_ expansion is stimulated by TNF-α, and patients with severe and progressive sarcoidosis have higher PB TNF-α levels than patients with milder and stable disease, providing a basis for escalating expansion of T_reg_ [27, 28]_._ However, upon binding to TNF-α, T_reg_ function is suppressed [29, 30] but their anti-proliferative function seems intact, which is hypothesized to explain the paradoxical PB lymphopenia despite dysfunctional T_regs_ [26]. Indeed, this is quite in keeping with that anti TNF-α therapy in patients with sarcoidosis led to increased PB lymphocyte counts [24, 25]. In this context, we think that our finding of an association with PB lymphopenia and HLA-DRB1*07 is interesting as genetic polymorphism of the TNF-α gene has been connected to *HLA-DRB1* variants and response to TNF-α inhibitor treatment in sarcoidosis [31, 32]. Thus, it is tempting to speculate that patients with lymphopenia and/ or HLA-DRB1*07 positive patients should receive 3^rd^ line treatment with TNF-α inhibitors early in disease course without first trying 1^st^ and 2^nd^ line treatments.

Strengths of this study include a well-defined patient group, composed of mainly white ancestry individuals of Nordic descent, all with non-LS and characterized both geno- and phenotypically. All data was collected in a register, by specialists in respiratory medicine, which should validate the diagnosis further.

As Karolinska University Hospital is a referral center, our data likely has a bias toward more severe cases. This can also explain the sex imbalance with more men included as men in Sweden seem to have a more severe disease than women [23]. There are also some other limitations. PB and BALF lymphocytes were not determined the same day, we did not actively screen for EPM, thus they may be under rated. The homogenous ethnic background of our cohort as we regard as a strength, in fact, can be a limitation, as the results may not be readily transferable to other ethnic populations.

To conclude, sarcoidosis patients with PB lymphopenia should be carefully monitored as they are at risk of developing a non-resolving disease and disclose a poor response to at least 1^st^ line treatment. The association with male sex and HLA-DRB1*07 may indicate an influence from sex hormones and a certain genotype on the sarcoidosis lymphopenia phenotype. Our results lend support to the hypothesis about sarcoidosis associated PB lymphopenia being secondary to depletion and not to sequestration in organs.

In the future, as we continue to include more patients and follow them longer, we plan to study more in detail lymphopenia, association with treatment responses and genes of importance for lymphopenia. Through the Mesargen network (www.mesargen.org), genotypic and phenotypic information is collected from local cohorts of sarcoidosis patients in several countries, enabling us to study genes and HLA-DRB1* alleles in relation to PB lymphopenia also in other ethnic groups.

## Data Availability

The data underlying this article will be shared on reasonable request to the corresponding author.

## Abbreviations

BALF: bronchoalveolar lavage fluid
HLA: human leukocyte antigen
LS: Löfgren´s syndrome
non-LS: non-Löfgren´s syndrome
PB: peripheral blood;
EPM: extra pulmonary manifestations
TRAV2.3: CD4+ T-cell receptor segment Vα2.3
WASOG: World Association of Sarcoidosis and other Granulomatous Disorders
